# HLA DRB1* Allele Lupus Erythematosus, Rheumatoid Arthritis, and other Autoimmune Disorders with Skin Involvement

**DOI:** 10.31138/mjr.111024.oad

**Published:** 2025-03-30

**Authors:** Eleni Klimi

**Affiliations:** Department of Dermatology, Thriassio General Hospital, Elefsina, Greece

**Keywords:** HLA, lupus, rheumatoid arthritis, DRB1, autoimmune

## Abstract

**Objective:**

A narrative short review has been made aiming to identify the most important immune disorders associated with the HLA DRB1*allele.

**Material and Methods:**

Data have been taken from PubMed, and 36 articles have been retrieved.

**Results:**

It has been found that DRB1*16:02 is associated with autoimmune diseases with production of autoantibodies, mainly systemic lupus erythematosus and myasthenia gravis. DRB1*04 is associated with lupus erythematosus, bullous pemphigoid, and pemphigus vulgaris. DRB1*is also associated with dermatitis herpetiformis, alopecia areata, acute generalised exanthematous pustulosis, and systemic sclerosis. No association of DRB1 has been found either with psoriasis or with psoriatic arthritis, while the association of Cw6 and DRB1*07 confers less severe joint damage in patients with psoriatic arthritis.

**Conclusion:**

DRB1* is associated with lupus erythematosus rheumatoid arthritis and with most immune disorders with skin involvement.

## INTRODUCTION

Within the Major Histocompatibility complex-MHC-, the HLA allele DRB1* is one of those most implicated in autoimmunity. The primary function of these glycoproteins is to regulate the immune response; in particular, class I and class II MHC molecules present peptides to CD8 cytotoxic T cells and to CD4 T cells, respectively.^1^ Susceptibility to autoimmune diseases has been consistently associated with MHC genotype, mostly MHC class II alleles. Several immune-mediated skin diseases have shown clear associations with specific HLA class II haplotypes, although the underlying mechanisms explaining how such HLA polymorphisms may confer susceptibility to certain diseases are still largely unknown.^2^ This article reports some of the immune disorders with skin involvement where the role of DRB1* seems prevalent.

## MATERIAL AND METHODS

**Figure 1.**
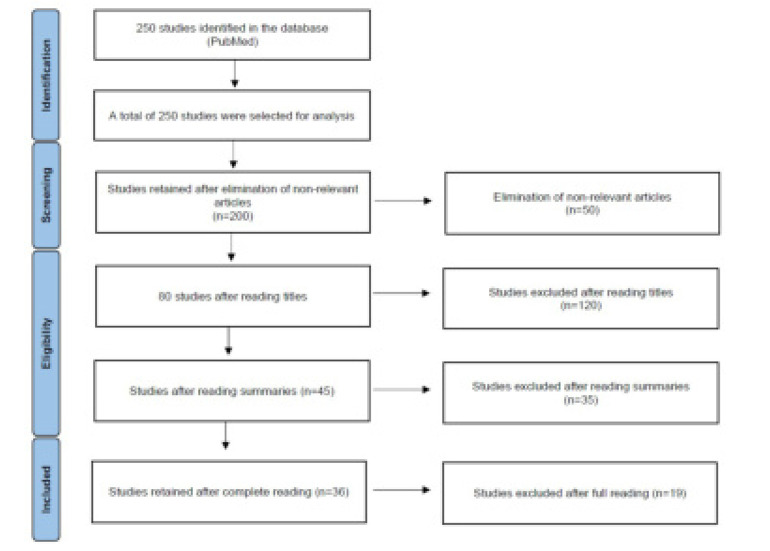
PRISMA flowchart.

The aim of this work is to review the current knowledge around HLA class II in autoimmune disorders associated with skin involvement. To achieve this objective, a PubMed search was conducted from September till October 2024, using the terms “HLA” and ‘’lupus erythematosus’’, or ‘’pemphigoid’’, or ‘’pemphigus vulgaris’’ and ‘’foliaceus’’, or ‘’dermatitis herpetiformis’’, or ‘’dermatomyositis’’, or ‘’epidermolysis bullosa acquisita’’, or ‘’rheumatoid arthritis’’, or ‘’psoriatic arthritis’’ and ‘’psoriasis’’, or ‘’alopecia areata’’, or ‘’acute generalized exanthematous pustulosis’’, or ‘’systemic sclerosis’’. Publication dates are within an interval of ten years, from 2013 until 2024. Exclusion criteria are included in the PRISMA flowchart (**Figure 1**).

## RESULTS

Systemic lupus erythematosus (SLE), a disease characterised with production of antibodies against self-antigens and consequently inflammatory events leading to the destruction of internal organs (kidney, heart, joints, brain, haemopoietic system), has been associated with HLADRB1* allele. DRB1*16:02 is associated with auto-immune disorders with production of autoantibodies including both SLE and myasthenia gravis (MG), while DRB1*14 is associated with late onset MG in Caucasians.^3^ In a group of Iranian patients with SLE, DRB1*03 has been significantly associated with production of autoantibodies anti-SSA/Ro (P_C_=0.02), anti-SSB/la (P_C_=0.002), and anticoagulant (P = 0.007).^4^ DRB1*15 has been associated with anti-SSA/Ro, DRB1*16 with anti-Sm, DRB1*04 with anti-nuclear antibodies in lupus (P = 0.032) and lupus nephritis (P = 0.001) in the Malay population of Malaysia.^5^ In a Japanese population, HLA DRB1*15:01 has been significantly associated with SLE (P = 5.1×10^−8^).^6^ DRB1*15:01 has also been the risk allele for SLE in European Americans, while DRB1*15:03 in African Americans (P = 7.0 × 10−25).^7^ Likewise, DRB1*15:01 is the allele most significantly associated with SLE and production of autoantibodies in Eastern Asians (P = 1.4x10^−27^, odds ratio (OR) = 1.57).^8^ DRB1 7:01 (P = 0.000236558) has been associated with high prevalence of bullous pemphigoid - a disease characterised with development of blisters on erythematous base - in Germans,^9^ and DRB1*1101 and DRB1*04 with bullous pemphigoid in Japanese patients (P < 0.05) for both alleles.^10^ HLA DRB1*14:54 alleles have been associated with mild pemphigus vulgaris (they were the most frequent alleles in both PV (20.5%) and PF (33.3%) patients), a disease characterised with blisters and erosions in Vietnamese patients.^11^ DRB1*04 and DRB1*14 are associated with pemphigus vulgaris in Turkey (P-value = .000), Syria (DRB1*402 subtype, P = 0.002) and India (OR, 27.85; 95% CI (confidence interval): 7.57–102.42).^12–14^ DRB1*04:02 has also been associated with pemphigus vulgaris in Brazilian and Argentinian patients.^15^ DRB1*01:02, DRB1*01, DRB1*4:02, DRB1*14:01 confer high susceptibility to pemphigus foliaceous in Brazil.^16^

Dermatitis herpetiformis – a disease with development of pruritic vesicles on erythematous base and with symptoms from the gastrointestinal tract - is associated with DRB103:01in a Chinese population (P = 2.1 × 10^−13^, OR = 9.7).^17^

Dermatomyositis, an immune disorder affecting the muscle and the skin, is associated with DRB1*3 01 (odds ratio (OR) = 1.66; 95% CI: 1.46–1.89; P = 1.4 × 10–14) and with DRB1*04, DRB1*12 (P = 0.01; pcorr NS; OR = 2.82; 95% CI: 1.15–6.76) (lung complication) and with DRB1*07 (P = 0.01; pcorr NS; OR = 4.78; 95% CI: 1.03–24.42) (dysphagia).^18–21^

Epidermolysis bullosa acquisita – a disorder characterised with skin fragility blisters and atrophic scars- is associated with HLA DRB1*15:03 in Black patients of African origin (P < 10[−3]).^22^

Rheumatoid arthritis - an inflammation of joints that may be accompanied with skin nodules, vasculitis and vasculopathy - has been associated with DRB1*01, DRB1*04:01, and DRB1*10 alleles.^23^

DRB1*alleles have not been associated with psoriatic arthritis in one study in France and in another in Spain. Cw6 and DRB1*07 together confer less severe joint damage in psoriatic arthritis.

Psoriasis limited to the skin is associated with DRB1*07 (relative risk, RR = 6.4, corrected P-value, Pc < or = 0.001) and DRB1*14:01 (age at onset < 40 years) (RR = 3.5, Pc < or = 0.001) alleles.^24,25^

Alopecia areata - a non-scarring alopecia whose clinical picture consists of hairless patches on the scalp or other parts of the body - is considered to be of autoimmune origin, as T lymphocytes surround the hair bulb in the affected individuals. HLA DRB1*04 [the OR (95% CIs) has been 1.49 (1.24–1.78) P<.01] and DRB1*16 [the OR (95% CIs) has been 1.61 (1.08–2.41) P<.01] have been associated with alopecia areata.^26^ HLA DRB1*1104 allele has also been found to be associated with early onset occurrence of alopecia areata (P = 0.004, RR = 2.1).^27^ A previous study has showed that adult patients affected with alopecia areata have a greater prevalence of psoriasis and rheumatoid arthritis.^28^ A recent study has revealed that adults who are first-degree family members of patients with alopecia areata have a greater prevalence of psoriasis, rheumatoid arthritis and alopecia areata.^29^ Development of psoriasis has been associated with DRB1*0701 allele but not with DRB1*04.

Acute generalised exanthematous pustulosis (AGEP) is a drug eruption characterised by development of sterile pustules on erythematous base induced by several antibiotics including ampicillin, macrolides, quinolones, metronidazole, and other drugs such as hydroxychloroquine. Although its mortality is quite low, involvement of internal organs may occur; therefore, it is considered a severe drug eruption. AGEP was also first associated with HLA DRB1*1101:04 thirty years ago by a French group of researchers. This finding has recently been confirmed in a Greek study that has included only one patient due to the rarity of the condition: AGEP affects 1 to 6 people out of a general population of 1 million.^30^

Finally, DRB1* has been associated with multiple sclerosis,^31^ particularly DRB1*08:04 (OR 3.2), DRB1*11:02 (OR 2.3) in African and European Americans,^32^ while association with HLA DRB1*15:02:01 (OR 95%) (Pc = 0.002) and DRB1*13:01:01 (OR 95%) (Pc = 1.055 × 10^−4^) is revealed in Thai patients.^33^ However, it is now believed that exogenous factors, such as exposure to silica, organic solvents, asbestos, and epoxy resins, play a much important role in the disease appearance than previously thought.^34^

## DISCUSSION

DRB1*04 allele has been associated with lupus erythematosus, bullous pemphigoid, pemphigus vulgaris, and pemphigus foliaceous, as well as dermatomyositis and systemic sclerosis. Rheumatoid arthritis, alopecia areata and psoriasis are found significantly more prevalent in patients with alopecia areata and in their family members, although only rheumatoid arthritis and alopecia areata are linked to DRB1*04 allele. However, literature search has revealed that the HLA DRB1*04 allele has recently diverted from the DRB1*0701 line-age after the separation of the human and chimpanzee species.^35^ This study highlights the role of DRB1* allele in numerous medical conditions and emphasises the economy of nature that uses the same tool in different circumstances, as stated by Kandel in his book “In Search of Memory”.^36^

## CONCLUSION

DRB1* allele has been found prevalent in most autoimmune diseases with skin involvement.

## CONFLICT OF INTEREST

The author declares no conflict of interest.

## FUNDING

None.

## References

[1] Sernicola A Mazzetto R Tartaglia J Ciolfi C Miceli P Alaibac M. Role of Human Leukocyte Antigen Class II in Antibody-Mediated Skin Disorders. Medicina (Kaunas) 2023 Nov 4;59(11):1950. doi: 10.3390/medicina59111950. PMID: 38003999. 38003999 PMC10673328

[2] Olbrich M Künstner A Witte M Busch H Fähnrich A. Genetics and Omics Analysis of Autoimmune Skin Blistering Diseases. Front Immunol 2019 Oct 15;10:2327. doi: 10.3389/fimmu.2019.02327. PMID: 31749790; PMCID: . 31749790 PMC6843061

[3] Klimi E Kataxaki E. Systemic Lupus Erythematosus and Myasthenia Gravis in a Male Patient: an HLA Case Investigated. Mediterr J Rheumatol 2021 Jun 27;32(3):285–6. doi: 10.31138/mjr.32.3.285. PMID: 34964036; PMCID: . 34964036 PMC8693297

[4] Rasouli-Saravani A Tahamoli-Roudsari A Basiri Z Babaei M Fazaeli A Roshanaei G Relevance of autoantibody profile with HLA-DRB1 and -DQB1 alleles in a group of Iranian systemic lupus erythematosus patients. Immunol Lett 2021 Sep;237:11–6. doi: 10.1016/j.imlet.2021.06.004. Epub 2021 Jun 26. PMID: 34186156. 34186156

[5] Selvaraja M Chin VK Abdullah M Arip M Amin-Nordin S. HLADRB1*04 as a Risk Allele to Systemic Lupus Erythematosus and Lupus Nephritis in the Malay Population of Malaysia. Front Med (Lausanne) 2021 Feb 10;7:598665. doi: 10.3389/fmed.2020.598665. PMID: 33644084; PMCID: . 33644084 PMC7902771

[6] Kawasaki A Kusumawati PA Kawamura Y Kondo Y Kusaoi M Amano H Genetic dissection of HLA-DRB1*15:01 and XL9 region variants in Japanese patients with systemic lupus erythematosus: primary role for HLA-DRB1*15:01. RMD Open 2023 May;9(2):e003214. doi: 10.1136/rmdopen-2023-003214. PMID: 37258043; PMCID: . 37258043 PMC10255040

[7] Hanscombe KB Morris DL Noble JA Dilthey AT Tombleson P Kaufman KM Genetic fine mapping of systemic lupus erythematosus MHC associations in Europeans and African Americans. Hum Mol Genet 2018 Nov 1;27(21):3813–24. doi: 10.1093/hmg/ddy280. PMID: 30085094; PMCID: . 30085094 PMC6196648

[8] Molineros JE Looger LL Kim K Okada Y Terao C Sun C Amino acid signatures of HLA Class-I and II molecules are strongly associated with SLE susceptibility and autoantibody production in Eastern Asians. PLoS Genet 2019 Apr 25;15(4):e1008092. doi: 10.1371/journal.pgen.1008092. PMID: 31022184; PMCID: . 31022184 PMC6504188

[9] Schwarm C Gola D Holtsche MM Dieterich A Bhandari A Freitag M German AIBD Study Group. Identification of two novel bullous pemphigoid- associated alleles, HLA-DQA1*05:05 and -DRB1*07:01, in Germans. Orphanet J Rare Dis 2021 May 19;16(1):228. doi: 10.1186/s13023-021-01863-9. PMID: 34011352; PMCID: . 34011352 PMC8136166

[10] Okazaki A Miyagawa S Yamashina Y Kitamura W Shirai T. Polymorphisms of HLA-DR and -DQ genes in Japanese patients with bullous pemphigoid. J Dermatol 2000 Mar;27(3):149–56. doi: 10.1111/j.1346-8138.2000.tb02141.x. PMID: 10774139. 10774139

[11] Le TTV Vuong TTB Ong TP Do MD. Allele frequency and the associations of HLA-DRB1 and HLA-DQB1 polymorphisms with pemphigus subtypes and disease severity. Medicine (Baltimore) 2022 Feb 18;101(7):e28855. doi: 10.1097/MD.0000000000028855. PMID: 35363186; PMCID: . 35363186 PMC9282124

[12] Dere G Yavuz IH Ozaydın Yavuz G Bayram Y Gunes Bilgili S Ozturk M. Assessment of HLA-A, HLA-DR, and HLA-DQ alleles in patients with pemphigus vulgaris from eastern of Turkey. J Cosmet Dermatol 2020 Sep;19(9):2432–7. doi: 10.1111/jocd.13298. Epub 2020 Jan 16. PMID: 31944522. 31944522

[13] Harfouch E Daoud S. Allelic variation in HLA-DRB1* loci in Syrian pemphigus vulgaris patients. Int J Dermatol 2014 Dec;53(12):1460–3. doi: 10.1111/ijd.12184. Epub 2014 Apr 2. PMID: 24697292. 24697292

[14] Priyadarshini A George R Daniel D Varughese S Jayaseelan V. Association between human leukocyte antigen-DRB1 and human leukocyte antigen-DQB1 alleles and pemphigus vulgaris in Indian patients: A case-control study. Indian J Dermatol Venereol Leprol 2018 May–Jun;84(3):280–4. doi: 10.4103/ijdvl.IJDVL_1014_16. PMID: 29582787. 29582787

[15] Weber R Monteiro F Preuhs-Filho G Rodrigues H Kalil J Miziara ID. HLA-DRB1*04:02, DRB1*08:04 and DRB1*14 alleles associated to pemphigus vulgaris in southeastern Brazilian population. Tissue Antigens 2011 Aug;78(2):92–3. doi: 10.1111/j.1399-0039.2011.01705.x. Epub 2011 May 9. PMID: 21554253. 21554253

[16] Brochado MJ Nascimento DF Campos W Deghaide NH Donadi EA Roselino AM. Differential HLA class I and class II associations in pemphigus foliaceus and pemphigus vulgaris patients from a prevalent Southeastern Brazilian region. J Autoimmun 2016 Aug;72:19–24. doi: 10.1016/j.jaut.2016.04.007. Epub 2016 May 10. PMID: 27178774. 27178774

[17] Sun Y Lin Y Yang B Wang C Fu X Bao F The HLA Alleles B*0801 and DRB1*0301 Are Associated with Dermatitis Herpetiformis in a Chinese Population. J Invest Dermatol 2016 Feb;136(2):530–2. doi: 10.1016/j.jid.2015.10.057. Epub 2015 Nov 18. PMID: 26967484. 26967484

[18] Deakin CT Bowes J Rider LG Miller FW Pachman LM Sanner H Juvenile Dermatomyositis Cohort and Biomarker Study, the Childhood Myositis Heterogeneity Study Group, and the Myositis Genetics Consortium (MYOGEN). Association with HLA-DRβ1 position 37 distinguishes juvenile dermatomyositis from adult-onset myositis. Hum Mol Genet 2022 Jul 21;31(14):2471–81. doi: 10.1093/hmg/ddac019. PMID: 35094092; PMCID: 35094092 PMC9307311

[19] Gao X Han L Yuan L Yang Y Gou G Sun H HLA class II alleles may influence susceptibility to adult dermatomyositis and polymyositis in a Han Chinese population. BMC Dermatol 2014 Jun 4;14:9. doi: 10.1186/1471-5945-14-9. PMID: 24894810; PMCID: . 24894810 PMC4062285

[20] Sherman MA Yang Q Gutierrez-Alamillo L Pak K Flegel WA Mammen AL Childhood Myositis Heterogeneity Collaborative Study Group. Clinical Features and Immunogenetic Risk Factors Associated With Additional Autoantibodies in Anti-Transcriptional Intermediary Factor 1γ Juvenile-Onset Dermatomyositis. Arthritis Rheumatol 2024 Apr;76(4):631–7. doi: 10.1002/art.42768. Epub 2024 Jan 30. PMID: 38059274; PMCID: . 38059274 PMC10965375

[21] DeWane ME Waldman R Lu J. Dermatomyositis: Clinical features and pathogenesis. J Am Acad Dermatol 2020 Feb;82(2):267–81. doi: 10.1016/j.jaad.2019.06.1309. Epub 2019 Jul 4. PMID: 31279808. 31279808

[22] Zumelzu C Le Roux-Villet C Loiseau P Busson M Heller M Aucouturier F Black patients of African descent and HLADRB1*15:03 frequency overrepresented in epidermolysis bullosa acquisita. J Invest Dermatol 2011 Dec;131(12):2386–93. doi: 10.1038/jid.2011.231. Epub 2011 Aug 11. PMID: 21833018. 21833018

[23] Srivastava S Rasool M. Genetics, epigenetics and autoimmunity constitute a Bermuda triangle for the pathogenesis of rheumatoid arthritis. Life Sci 2024 Sep 26;357:123075. doi: 10.1016/j.lfs.2024.123075. Epub ahead of print. PMID: 39341491. 39341491

[24] Massy E Pedini P Pollet E Martin M Roudier J Picard C Association study between HLA-A, -B, -C, -DRB1 alleles and Psoriatic arthritis in southern France. Hum Immunol 2022 Jun;83(6):515–20. doi: 10.1016/j.humimm.2022.04.001. Epub 2022 Apr 12. PMID: 35428536 35428536

[25] Jee SH Tsai TF Tsai WL Liaw SH Chang CH Hu CY. HLADRB1*0701 and DRB1*1401 are associated with genetic susceptibility to psoriasis vulgaris in a Taiwanese population. Br J Dermatol 1998 Dec;139(6):978–83. doi: 10.1046/j.1365-2133.1998.02552.x. PMID: 9990359. 9990359

[26] Ji C Liu S Zhu K Luo H Li Q Zhang Y HLA-DRB1 polymorphisms and alopecia areata disease risk: A systematic review and meta-analysis. Medicine (Baltimore) 2018 Aug;97(32):e11790. doi: 10.1097/MD.0000000000011790. PMID: 30095639; PMCID: . 30095639 PMC6133534

[27] Marques Da Costa C Dupont E Van der Cruys M Andrien M Hidajat M Song M Earlier occurrence of severe alopecia areata in HLA-DRB1*11-positive patients. Dermatology 2006;213(1):12–4. doi: 10.1159/000092831. PMID: 16778420. 16778420

[28] Chu SY Chen YJ Tseng WC Lin MW Chen TJ Hwang CY Comorbidity profiles among patients with alopecia areata: the importance of onset age, a nationwide population-based study. J Am Acad Dermatol 2011 Nov;65(5):949–56. doi: 10.1016/j.jaad.2010.08.032. Epub 2011 May 25. PMID: 21616562. 21616562

[29] Klimi E. Family history of autoimmunity and endocrine disorders in patients with alopecia areata. A Greek study. Int J Trichol 2022 Sep–Oct;14(5):186–7. 10.4103/ijt.ijt_120_21 PMC9674061 36404888

[30] Klimi E. Acute generalized exanthematous pustulosis induced by hydroxychloroquine associated with human leukocyte antigen-B51 (HLA-B51), DRB1*1101:04, DQ03,05, and for the first time in the literature with HLA-B15. Turk Arch Dermatol Venereol 2020;54:69–70.

[31] Xu Y Mo N Jiang Z Lu S Fu S Wei X Human leukocyte antigen (HLA)-DRB1 allele polymorphisms and systemic sclerosis. Mod Rheumatol 2019 Nov;29(6):984–91. doi: 10.1080/14397595.2018.1519148. Epub 2019 Mar 25. PMID: 30175673. 30175673

[32] Gourh P Safran SA Alexander T Boyden SE Morgan ND Shah AA HLA and autoantibodies define scleroderma subtypes and risk in African and European Americans and suggest a role for molecular mimicry. Proc Natl Acad Sci U S A 2020 Jan 7;117(1):552–62. doi: 10.1073/pnas.1906593116. Epub 2019 Dec 23. PMID: 31871193; PMCID: . 31871193 PMC6955366

[33] Louthrenoo W Kasitanon N Wongthanee A Okudaira Y Takeuchi A Noguchi H HLA Association among Thai Patients with Diffuse and Limited Cutaneous Systemic Sclerosis. Biomedicines 2024 Jun 18;12(6):1347. doi: 10.3390/biomedicines12061347. PMID: 38927554; PMCID: . 38927554 PMC11201995

[34] Ouchene L Muntyanu A Lavoué J Baron M Litvinov IV Netchiporouk E. Toward Understanding of Environmental Risk Factors in Systemic Sclerosis. [Formula: see text]. J Cutan Med Surg 2021 Mar–Apr;25(2):188–204. doi: 10.1177/1203475420957950. Epub 2020 Sep 28. PMID: 32988228. 32988228

[35] Hohjoh H Ohashi J Takasu M Nishioka T Ishida T Tokunaga K. Recent divergence of the HLA-DRB1*04 allelic lineage from the DRB1*0701 lineage after the separation of the human and chimpanzee species. Immunogenetics 2003 Mar;54(12):856–61. 12671736 10.1007/s00251-003-0539-z

[36] Kandel ER. In search of a memory: The emergence of a New Science of Mind. 2006. W.W. Norton & Company.: New York, New York, USA.352p.

